# Supplementary material for: The Best of Both Worlds: A Framework for Combining Degradation Prediction with High Performance Super-Resolution Networks
Source: Sensors (Basel). 2022 Dec 30;23(1):419. doi: 10.3390/s23010419 (PMC9823731; doi:10.3390/s23010419)
Supplement: Supplementary file 1 [file sensors-23-00419-s001.zip › sensors-2053450-supplementary.pdf]

Supplementary Material for 'The Best of Both Worlds: a Framework for Combining Degradation Prediction with High Performance Super-Resolution Networks'

Matthew Aquilina, Keith George Ciantar, Christian Galea, Kenneth P. Camilleri, Reuben A. Farrugia, John Abela

This document contains a number of supplementary results and figures which support the main text. Please refer to the main manuscript for further details.

Simple Pipeline Results

Table S1: SSIM SR results corresponding to Table 1 from the main paper. 'low' refers to a σ of 0.2, 'med' refers to a σ of 1.6 and 'high' refers to a σ of 3.0. The best result for each set is shown in red, while the second-best result is shown in blue.

**Model**	**Set5**			**Set14**			**BSDS100**			**Manga109**			**Urban100**		
	**low**	**med**	**high**	**low**	**med**	**high**	**low**	**med**	**high**	**low**	**med**	**high**	**low**	**med**	**high**
**Baselines**															
Bicubic	0.7909	0.7513	0.6736	0.6803	0.6384	0.5700	0.6452	0.6058	0.5458	0.7716	0.7323	0.6659	0.6376	0.5941	0.5240
Lanczos	0.7986	0.7625	0.6814	0.6888	0.6487	0.5761	0.6534	0.6149	0.5510	0.7775	0.7418	0.6720	0.6452	0.6044	0.5302
**Non-Blind**															
RCAN	0.8830	0.8783	0.8564	0.7664	0.7632	0.7314	0.7217	0.7170	0.6897	0.9015	0.9000	0.8728	0.7834	0.7760	0.7357
MA	0.8845	0.8837	0.8615	0.7671	0.7669	0.7408	0.7221	0.7212	0.6953	0.9041	0.9047	0.8857	0.7849	0.7812	0.7479
MA (all)	0.8836	0.8828	0.8620	0.7667	0.7670	0.7407	0.7216	0.7213	0.6958	0.9036	0.9051	0.8863	0.7837	0.7808	0.7483
MA (PCA)	0.8846	0.8834	0.8617	0.7677	0.7667	0.7401	0.7224	0.7214	0.6951	0.9040	0.9048	0.8852	0.7852	0.7812	0.7475
SRMD	0.8844	0.8836	0.8622	0.7673	0.7670	0.7406	0.7222	0.7212	0.6956	0.9039	0.9048	0.8855	0.7844	0.7809	0.7477
SFT	0.8847	0.8832	0.8627	0.7677	0.7671	0.7406	0.7228	0.7215	0.6957	0.9043	0.9052	0.8863	0.7859	0.7817	0.7482
DA	0.8845	0.8836	0.8628	0.7676	0.7673	0.7410	0.7221	0.7215	0.6957	0.9040	0.9049	0.8861	0.7846	0.7816	0.7484
DA (all)	0.8847	0.8837	0.8622	0.7666	0.7667	0.7401	0.7217	0.7211	0.6957	0.9033	0.9050	0.8856	0.7822	0.7807	0.7480
DGFMB	0.8845	0.8836	0.8623	0.7672	0.7669	0.7406	0.7224	0.7216	0.6956	0.9041	0.9048	0.8860	0.7850	0.7812	0.7485
DGFMB (no FC)	0.8849	0.8834	0.8625	0.7673	0.7669	0.7406	0.7224	0.7211	0.6954	0.9040	0.9046	0.8856	0.7851	0.7810	0.7479

Table S2: SSIM SR results corresponding to Table 3 from the main paper. 'low' refers to a σ of 0.2, 'med' refers to a σ of 1.6 and 'high' refers to a σ of 3.0. The best result for each set is shown in red, while the second-best result is shown in blue.

Model	Set5			Set14			BSDS100			Manga109			Urban100		
	low	med	high	low	med	high	low	med	high	low	med	high	low	med	high
**Classical**															
Bicubic	0.7909	0.7513	0.6736	0.6803	0.6384	0.5700	0.6452	0.6058	0.5458	0.7716	0.7323	0.6659	0.6376	0.5941	0.5240
Lanczos	0.7986	0.7625	0.6814	0.6888	0.6487	0.5761	0.6534	0.6149	0.5510	0.7775	0.7418	0.6720	0.6452	0.6044	0.5302
**Pretrained**															
IKC (pretrained-best-iter)	0.8832	0.8751	0.8604	0.7658	0.7486	0.7382	0.7214	0.7083	0.6912	0.9019	0.8763	0.8612	0.7769	0.7488	0.7262
IKC (pretrained-last-iter)	0.8827	0.8663	0.8585	0.7675	0.7436	0.7269	0.7233	0.6967	0.6872	0.9015	0.8694	0.8454	0.7754	0.7389	0.7149
DASR (pretrained)	0.8797	0.8747	0.8539	0.7617	0.7512	0.7205	0.7177	0.7087	0.6796	0.8960	0.8909	0.8690	0.7665	0.7521	0.7182
DANv1 (pretrained)	0.8828	0.8787	0.8610	0.7657	0.7594	0.7331	0.7211	0.7144	0.6877	0.9011	0.9007	0.8839	0.7793	0.7706	0.7401
DANv2 (pretrained)	0.8849	0.8812	0.8589	0.7678	0.7617	0.7329	0.7233	0.7171	0.6868	0.9028	0.9009	0.8827	0.7850	0.7736	0.7395
**Non-Blind**															
RCAN-MA (true sigma)	0.8845	0.8837	0.8615	0.7671	0.7669	0.7408	0.7221	0.7212	0.6953	0.9041	0.9047	0.8857	0.7849	0.7812	0.7479
HAN-MA (true sigma)	0.8842	0.8831	0.8611	0.7671	0.7670	0.7407	0.7222	0.7213	0.6956	0.9043	0.9050	0.8862	0.7850	0.7814	0.7483
RCAN-MA (noisy sigma)	0.8839	0.8808	0.8589	0.7669	0.7639	0.7328	0.7223	0.7177	0.6902	0.9025	0.9010	0.8754	0.7825	0.7741	0.7343
**RCAN/DAN**															
DANv1	0.8791	0.8749	0.8511	0.7608	0.7569	0.7261	0.7170	0.7120	0.6840	0.8921	0.8911	0.8652	0.7620	0.7550	0.7187
DANv1 (cosine)	0.8820	0.8774	0.8540	0.7638	0.7604	0.7305	0.7195	0.7149	0.6870	0.8980	0.8961	0.8720	0.7721	0.7642	0.7269
RCAN (batch size 4)	0.8830	0.8792	0.8564	0.7662	0.7636	0.7329	0.7221	0.7174	0.6900	0.9019	0.9007	0.8760	0.7816	0.7743	0.7351
RCAN-DAN	0.8840	0.8796	0.8583	0.7669	0.7644	0.7323	0.7223	0.7183	0.6902	0.9027	0.9019	0.8776	0.7828	0.7754	0.7360
RCAN-DAN (sigma)	0.8838	0.8807	0.8591	0.7676	0.7654	0.7328	0.7229	0.7193	0.6908	0.9029	0.9019	0.8775	0.7811	0.7757	0.7356
**HAN/DAN**															
HAN (batch size 4)	0.8837	0.8795	0.8550	0.7668	0.7636	0.7316	0.7221	0.7175	0.6895	0.9017	0.9003	0.8739	0.7822	0.7743	0.7321
HAN-DAN	0.8842	0.8797	0.8579	0.7671	0.7638	0.7331	0.7225	0.7172	0.6894	0.9027	0.9010	0.8773	0.7829	0.7751	0.7354
**RCAN/Contrastive**															
RCAN (batch size 8)	0.8830	0.8783	0.8564	0.7664	0.7632	0.7314	0.7217	0.7170	0.6897	0.9015	0.9000	0.8728	0.7834	0.7760	0.7357
RCAN-MoCo	0.8844	0.8801	0.8577	0.7671	0.7629	0.7332	0.7221	0.7174	0.6904	0.9022	0.9008	0.8770	0.7819	0.7742	0.7343
RCAN-SupMoCo	0.8839	0.8804	0.8578	0.7671	0.7630	0.7329	0.7227	0.7177	0.6901	0.9030	0.9010	0.8771	0.7831	0.7748	0.7356
RCAN-regression	0.8835	0.8795	0.8559	0.7668	0.7640	0.7331	0.7229	0.7180	0.6899	0.9024	0.9005	0.8772	0.7825	0.7736	0.7337
RCAN-SupMoCo-regression	0.8845	0.8805	0.8567	0.7667	0.7645	0.7321	0.7223	0.7177	0.6905	0.9031	0.9016	0.8770	0.7831	0.7749	0.7348
RCAN-WeakCon	0.8837	0.8794	0.8582	0.7673	0.7641	0.7334	0.7229	0.7189	0.6905	0.9027	0.9009	0.8760	0.7826	0.7747	0.7350
RCAN-SupMoCo (online)	0.8836	0.8799	0.8591	0.7673	0.7634	0.7245	0.7227	0.7186	0.6903	0.9030	0.9005	0.8763	0.7826	0.7743	0.7317
RCAN-SupMoCo (ResNet)	0.8829	0.8789	0.8570	0.7666	0.7630	0.7292	0.7221	0.7170	0.6882	0.9017	0.8998	0.8629	0.7798	0.7714	0.7250
**HAN/Contrastive**															
HAN (batch size 8)	0.8836	0.8802	0.8554	0.7669	0.7638	0.7311	0.7223	0.7180	0.6896	0.9025	0.9013	0.8747	0.7826	0.7754	0.7337
HAN-SupMoCo-regression	0.8835	0.8801	0.8581	0.7665	0.7634	0.7332	0.7220	0.7167	0.6899	0.9019	0.8999	0.8774	0.7815	0.7735	0.7348
**Extensions**															
RCAN-DAN (pretrained estimator)	0.8839	0.8794	0.8576	0.7678	0.7647	0.7332	0.7227	0.7185	0.6908	0.9034	0.9024	0.8769	0.7837	0.7762	0.7363
RCAN (batch size 8, long-term)	0.8836	0.8806	0.8580	0.7670	0.7642	0.7325	0.7222	0.7178	0.6902	0.9027	0.9014	0.8757	0.7834	0.7760	0.7357
RCAN-SupMoCo-regression (long-term)	0.8842	0.8797	0.8570	0.7675	0.7649	0.7333	0.7231	0.7188	0.6913	0.9037	0.9021	0.8786	0.7852	0.7771	0.7377

Table S3: LPIPS results (smaller is better) for Real-ESRGAN models tested. The datasets are identical to those used for the simple pipeline analysis. The best result for each set is shown in red, while the second-best result is shown in blue.

Model	Set5			Set14			BSDS100			Manga109			Urban100		
	low	med	high	low	med	high	low	med	high	low	med	high	low	med	high
Bicubic	0.1131	0.1582	0.2543	0.1672	0.2134	0.3052	0.2040	0.2465	0.3300	0.1398	0.1905	0.2900	0.2117	0.2594	0.3501
Lanczos	0.1051	0.1458	0.2442	0.1607	0.2024	0.2956	0.1992	0.2370	0.3216	0.1307	0.1781	0.2798	0.2057	0.2495	0.3409
Real-ESRGAN (batch size 4)	0.0211	0.0266	0.0468	0.0433	0.0490	0.0738	0.0555	0.0611	0.0878	0.0210	0.0225	0.0340	0.0492	0.0535	0.0789
Real-ESRGAN (batch size 8)	0.0250	0.0299	0.0440	0.0447	0.0502	0.0792	0.0553	0.0624	0.0872	0.0208	0.0231	0.0368	0.0525	0.0585	0.0831
Real-ESRGAN (MA-non-blind)	0.0200	0.0203	0.0271	0.0418	0.0421	0.0535	0.0518	0.0540	0.0650	0.0181	0.0177	0.0241	0.0463	0.0480	0.0593
Real-ESRGAN (SupMoCo-regression)	0.0227	0.0302	0.0416	0.0478	0.0532	0.0899	0.0607	0.0661	0.0815	0.0213	0.0241	0.0327	0.0529	0.0543	0.0767
Real-ESRGAN-DAN	0.0185	0.0275	0.0392	0.0421	0.0465	0.0663	0.0491	0.0578	0.0833	0.0196	0.0217	0.0289	0.0458	0.0509	0.0729

Table S4: PSNR (dB) results for ELAN models tested. The datasets are identical to those used for the simple pipeline analysis. ELAN (MA-all-non-blind) refers to an ELAN model upgraded with a single meta-attention block, with its attention vector multiplied with the network feature map at 36 points through the network (one for each attention block). The best result for each set is shown in red, while the second-best result is shown in blue.

Model	Set5			Set14			BSDS100			Manga109			Urban100		
	low	med	high	low	med	high	low	med	high	low	med	high	low	med	high
Bicubic	27.084	25.857	23.867	24.532	23.695	22.286	24.647	23.998	22.910	23.608	22.564	20.932	21.805	21.104	19.944
Lanczos	27.462	26.210	24.039	24.760	23.925	22.409	24.811	24.173	23.007	23.923	22.850	21.071	21.989	21.293	20.046
ELAN	27.341	27.400	24.759	24.813	24.715	22.914	24.815	24.789	23.403	24.233	23.894	21.648	22.051	21.982	20.477
ELAN (MA-non-blind)	27.401	27.391	24.731	24.841	24.714	22.899	24.841	24.788	23.396	24.255	23.883	21.629	22.077	21.977	20.465
ELAN (MA-all-non-blind)	30.431	30.405	29.007	26.733	26.728	25.922	26.156	26.177	25.541	28.539	28.764	27.545	24.590	24.546	23.849

Table S5: SSIM results for ELAN models tested. The datasets are identical to those used for the simple pipeline analysis. ELAN (MA-all-non-blind) refers to an ELAN model upgraded with a single meta-attention block, with its attention vector multiplied with the network feature map at 36 points through the network (one for each attention block). The best result for each set is shown in red, while the second-best result is shown in blue.

Model	Set5			Set14			BSDS100			Manga109			Urban100		
	low	med	high	low	med	high	low	med	high	low	med	high	low	med	high
Bicubic	0.7909	0.7513	0.6736	0.6803	0.6384	0.5700	0.6452	0.6058	0.5458	0.7716	0.7323	0.6659	0.6376	0.5941	0.5240
Lanczos	0.7986	0.7625	0.6814	0.6888	0.6487	0.5761	0.6534	0.6149	0.5510	0.7775	0.7418	0.6720	0.6452	0.6044	0.5302
ELAN	0.7962	0.7933	0.7078	0.7074	0.6856	0.5986	0.6772	0.6512	0.5709	0.7805	0.7697	0.6914	0.6597	0.6405	0.5528
ELAN (MA-non-blind)	0.7970	0.7932	0.7071	0.7079	0.6857	0.5981	0.6775	0.6508	0.5702	0.7814	0.7703	0.6913	0.6610	0.6411	0.5530
ELAN (MA-all-non-blind)	0.8778	0.8773	0.8529	0.7591	0.7585	0.7311	0.7142	0.7133	0.6879	0.8887	0.8895	0.8664	0.7669	0.7637	0.7336

Complex Pipeline Results

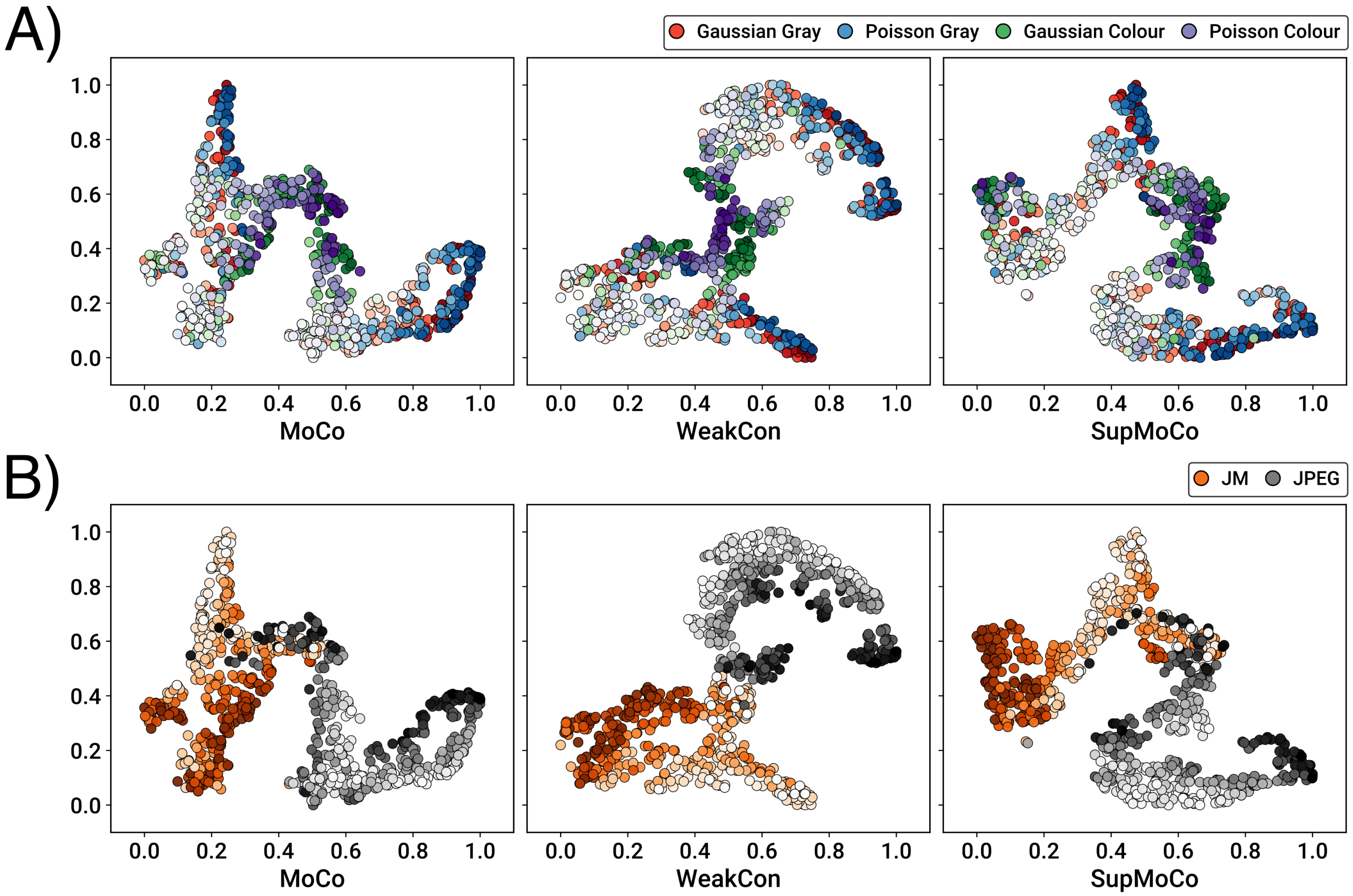

Figure S1: t-SNE plots (perplexity value of 40) showing the separation power of the different contrastive learning algorithms considered on the complex pipeline. All models were evaluated at epoch 2000. The test dataset consisted of 2472 images (8 sets of 309 images of BSDS100/Manga109/Urban100) degraded using the full complex pipeline (random degradation parameters). Only 800 images (randomly selected) are plotted per panel, to reduce cluttering. Each dimension was independently normalised in the range $[0, 1]$ after computing the t-SNE results. A) t-SNE plots with each image coloured according to the noise applied. The colour intensity of each point corresponds to the noise magnitude (in the range $[1, 30]$ for Gaussian noise and $[0.05, 3]$ for Poisson noise). All three encoders are capable of separating noise magnitudes, but lose the ability to distinctly separate the two noise classes. B) t-SNE plots with each image coloured according to the compression applied. The colour intensity of each point corresponds to the compression level, which is in the range $[30, 95]$ (higher is better) for JPEG and $[20, 40]$ (lower is better) for JM H.264. All three encoders are capable of separating compression magnitudes, but struggle to separate the two compression types when the compression is very low.

Table S6: SSIM SR results corresponding to Table 6 from the main paper. Please refer to Table 4 in the main paper for full details on each testing scenario. The best result for each set is shown in red, while the second-best result is shown in blue.

Dataset	Model	JPEG	JM	Poisson	Gaussian	Iso	Aniso	Iso + Gaussian	Gaussian + JPEG	Iso + Gaussian + JPEG	Aniso + Poisson + JM	Iso/Aniso + Gaussian/Poisson + JPEG/JM
	Bicubic	0.5777	0.5870	0.4443	0.4341	0.5880	0.6075	0.3847	0.4284	0.3932	0.4229	0.4067
	Lanczos	0.5792	0.5911	0.4283	0.4173	0.5964	0.6165	0.3674	0.4133	0.3787	0.4090	0.3922
	RCAN (batch size 4)	0.6117	0.6213	0.6004	0.5995	0.6660	0.6744	0.5625	0.5749	0.5465	0.5543	0.5503
	RCAN (batch size 8)	0.6117	0.6211	0.6014	0.5991	0.6649	0.6723	0.5617	0.5751	0.5467	0.5548	0.5505
	RCAN (non-blind)	N/A	N/A	N/A	N/A	N/A	N/A	N/A	N/A	0.5482	0.5494	0.5496
	RCAN (non-blind, no blur)	N/A	N/A	N/A	N/A	N/A	N/A	N/A	0.5788	0.5480	0.5512	0.5503
	RCAN (non-blind, no noise)	N/A	N/A	N/A	N/A	N/A	N/A	N/A	N/A	0.5483	0.5542	0.5519
BSDS100	RCAN (non-blind, no compression)	N/A	N/A	N/A	N/A	N/A	N/A	0.5629	N/A	0.5491	0.5526	0.5499
	RCAN (MoCo)	0.6108	0.6217	0.6017	0.6002	0.6590	0.6699	0.5619	0.5750	0.5469	0.5561	0.5511
	RCAN (WeakCon)	0.6115	0.6207	0.5995	0.5942	0.6614	0.6709	0.5568	0.5754	0.5468	0.5557	0.5508
	RCAN (SupMoCo)	0.6106	0.6220	0.5968	0.5949	0.6612	0.6733	0.5569	0.5754	0.5464	0.5550	0.5504
	RCAN (SupMoCo, all)	0.6119	0.6225	0.6009	0.6001	0.6655	0.6740	0.5617	0.5755	0.5465	0.5554	0.5507
	RCAN-DAN	0.6127	0.6229	0.6030	0.6004	0.6657	0.6759	0.5632	0.5754	0.5470	0.5553	0.5508
	RCAN (trained on simple pipeline)	0.5742	0.6000	0.3166	0.2976	0.7145	0.6953	0.2492	0.3197	0.2929	0.2979	0.2921
	Bicubic	0.7041	0.7315	0.4972	0.5311	0.7131	0.7337	0.4783	0.5390	0.5001	0.4821	0.4898
	Lanczos	0.7034	0.7347	0.4793	0.5135	0.7223	0.7428	0.4607	0.5236	0.4858	0.4642	0.4735
	RCAN (batch size 4)	0.8093	0.8173	0.7959	0.8028	0.8389	0.8446	0.7623	0.7777	0.7418	0.7468	0.7438
	RCAN (batch size 8)	0.8093	0.8182	0.7958	0.8017	0.8356	0.8397	0.7613	0.7784	0.7427	0.7467	0.7441
	RCAN (non-blind)	N/A	N/A	N/A	N/A	N/A	N/A	N/A	N/A	0.7461	0.7469	0.7466
	RCAN (non-blind, no blur)	N/A	N/A	N/A	N/A	N/A	N/A	N/A	0.7850	0.7443	0.7483	0.7469
	RCAN (non-blind, no noise)	N/A	N/A	N/A	N/A	N/A	N/A	N/A	N/A	0.7438	0.7453	0.7444
Manga109	RCAN (non-blind, no compression)	N/A	N/A	N/A	N/A	N/A	N/A	0.7657	N/A	0.7477	0.7476	0.7470
	RCAN (MoCo)	0.8072	0.8174	0.7947	0.8012	0.8268	0.8358	0.7617	0.7767	0.7419	0.7465	0.7436
	RCAN (WeakCon)	0.8078	0.8177	0.7879	0.7901	0.8295	0.8370	0.7464	0.7758	0.7415	0.7465	0.7433
	RCAN (SupMoCo)	0.8096	0.8193	0.7889	0.7999	0.8294	0.8380	0.7594	0.7793	0.7418	0.7471	0.7438
	RCAN (SupMoCo, all)	0.8091	0.8192	0.7922	0.8015	0.8309	0.8377	0.7575	0.7793	0.7409	0.7464	0.7432
	RCAN-DAN	0.8111	0.8212	0.7959	0.8024	0.8398	0.8438	0.7626	0.7795	0.7442	0.7485	0.7456
	RCAN (trained on simple pipeline)	0.7012	0.7575	0.3702	0.3997	0.8975	0.8600	0.3368	0.4332	0.3998	0.3266	0.3581
	Bicubic	0.5791	0.5964	0.4485	0.4372	0.5737	0.5969	0.3804	0.4321	0.3868	0.4189	0.4014
	Lanczos	0.5809	0.6016	0.4367	0.4241	0.5834	0.6069	0.3671	0.4206	0.3759	0.4083	0.3904
	RCAN (batch size 4)	0.6696	0.6842	0.6625	0.6614	0.7006	0.7086	0.6077	0.6329	0.5861	0.6074	0.5957
	RCAN (batch size 8)	0.6692	0.6834	0.6629	0.6601	0.6985	0.7063	0.6071	0.6340	0.5872	0.6071	0.5963
	RCAN (non-blind)	N/A	N/A	N/A	N/A	N/A	N/A	N/A	N/A	0.5920	0.6074	0.5983
	RCAN (non-blind, no blur)	N/A	N/A	N/A	N/A	N/A	N/A	N/A	0.6377	0.5885	0.6084	0.5971
	RCAN (non-blind, no noise)	N/A	N/A	N/A	N/A	N/A	N/A	N/A	N/A	0.5883	0.6059	0.5953
Urban100	RCAN (non-blind, no compression)	N/A	N/A	N/A	N/A	N/A	N/A	0.6122	N/A	0.5929	0.6088	0.6000
	RCAN (MoCo)	0.6686	0.6831	0.6626	0.6594	0.6886	0.7017	0.6052	0.6340	0.5874	0.6084	0.5968
	RCAN (WeakCon)	0.6693	0.6836	0.6617	0.6511	0.6941	0.7037	0.5909	0.6348	0.5872	0.6080	0.5966
	RCAN (SupMoCo)	0.6684	0.6834	0.6571	0.6538	0.6912	0.7042	0.5987	0.6343	0.5863	0.6079	0.5960
	RCAN (SupMoCo, all)	0.6676	0.6824	0.6592	0.6563	0.6968	0.7053	0.6005	0.6324	0.5852	0.6070	0.5952
	RCAN-DAN	0.6719	0.6870	0.6657	0.6632	0.7027	0.7109	0.6094	0.6357	0.5891	0.6099	0.5985
	RCAN (trained on simple pipeline)	0.5897	0.6481	0.3616	0.3396	0.7688	0.7395	0.2733	0.3490	0.3067	0.3216	0.3105

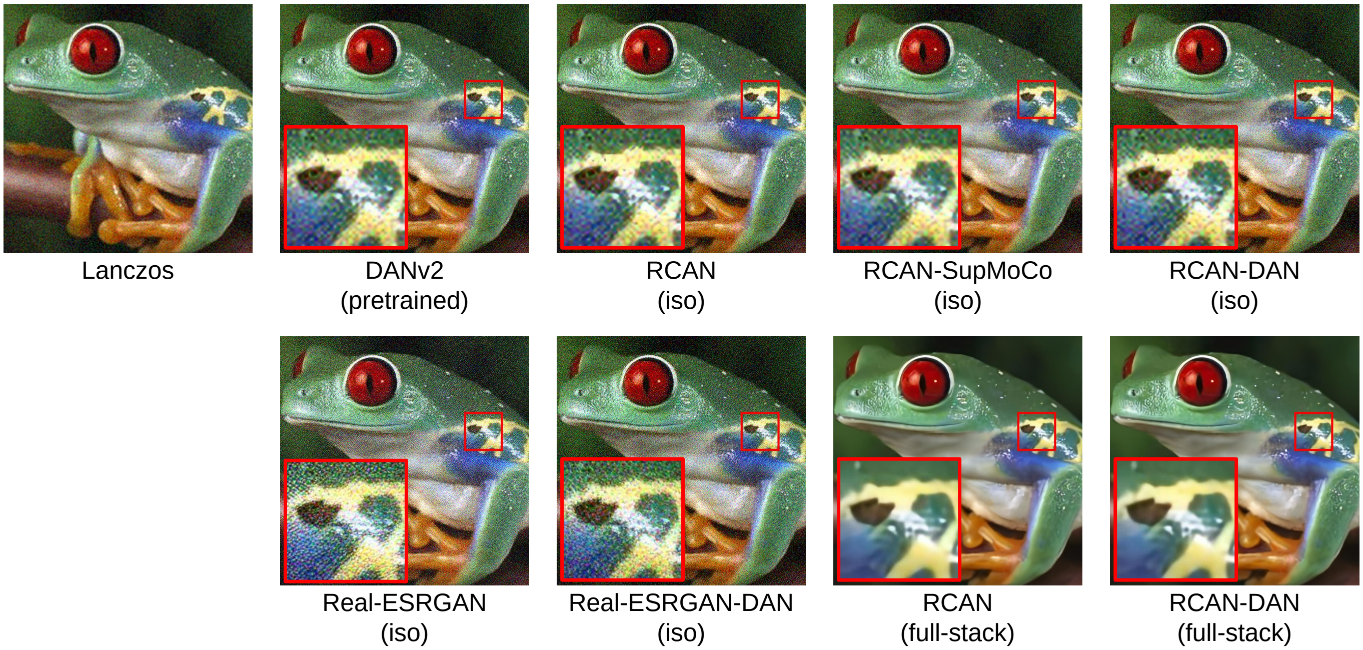

Figure S2: Comparison of the SR results of various models on the frog image from RealSRSet. All simple pipeline models (marked as iso) are incapable of removing the noise from the image. The complex pipeline models (marked as full-stack) produce significantly improved results.
